# Efficacy of powdered alfalfa leaves to ameliorate the toxic effects of aflatoxin B_1_ in turkey poults

**DOI:** 10.1007/s12550-024-00527-4

**Published:** 2024-02-29

**Authors:** M. J. Nava-Ramírez, J. A. Maguey-González, S. Gómez-Rosales, J. O. Hernández-Ramírez, J. D. Latorre, Xiangwei Du, C. López-Coello, B. M. Hargis, G. Téllez-Isaías, A. Vázquez-Durán, A. Méndez-Albores

**Affiliations:** 1 Unidad de Investigación Multidisciplinaria L14 (Alimentos, Micotoxinas, y Micotoxicosis), Facultad de Estudios Superiores Cuautitlán, Universidad Nacional Autónoma de México (UNAM), Cuautitlán Izcalli, 54714 Mexico; 2 Department of Poultry Science, University of Arkansas, Fayetteville, AR 72701 USA; 3 Centro Nacional de Investigación Disciplinaria en Fisiología y Mejoramiento Animal (CENID-INIFAP), Km 1 Carretera a Colon Ajuchitlán, Querétaro, 76280 Mexico; 4 Veterinary Medical Diagnostic Laboratory, College of Veterinary Medicine, University of Missouri, Columbia, MO 65211 USA; 5 Departamento de Medicina y Zootecnia de Aves, Facultad de Medicina Veterinaria y Zootecnia, Universidad Nacional Autónoma de México (UNAM), Ciudad de Mexico, 04510 Mexico

**Keywords:** Turkey poults, Aflatoxin B_1_, Powdered alfalfa leaves, Adsorption, Performance, Histology

## Abstract

This experiment was conducted to determine the effect of an adsorbent material based on powdered alfalfa leaves added in the aflatoxin B_1_ (AFB_1_)-contaminated diet of turkey poults on production parameters, blood cell count, serum biochemistry, liver enzymes, and liver histology. For this purpose, three hundred and fifty female Nicholas-700 poults were randomly assigned into five treatments: (1) Control, AFB_1_-free diet; (2) AF, diet contaminated with 250 ng AFB_1_/g; (3) Alfalfa, AFB_1_-free diet + 0.5% (w/w) adsorbent; (4) AF+alfalfa, diet contaminated with 250 ng AFB_1_/g + 0.5% (w/w) adsorbent, and (5) AF+ yeast cell wall (YCW), diet contaminated with 250 ng AFB_1_/g + 0.5% (w/w) of yeast cell wall (a commercial mycotoxin binder used as reference material). The in vivo efficacy of powdered alfalfa leaves was assessed during a 28-day period. In general, the addition of powdered alfalfa leaves in the AFB_1_-free diet gave the best performance results (body weight, body weight gain, and feed intake) and improved the values of total protein, glucose, calcium, creatinine, and blood urea nitrogen. Moreover, the addition of powdered alfalfa leaves in the AFB_1_-contaminated diet enhanced body weight and body weight gain and significantly reduced the feed intake, compared to the AF and AF+YCW groups. Additionally, significant alterations in serum parameters were observed in poults intoxicated with the AFB_1_, compared to the Control group. Furthermore, typical histopathological lesions were observed in the liver of the AF group, which were significantly ameliorated with the addition of powdered alfalfa leaves. Conclusively, these results pointed out that low inclusion of powdered alfalfa leaves in the contaminated feed counteracted the adverse effects of AFB_1_ in turkey poults.

## Introduction

Aflatoxins are mycotoxins composed of a pentacyclic structure with a difuran and coumarin skeleton and are mainly produced by fungal species of the genus *Aspergillus* (Lala et al. 2016). Aflatoxin B_1_ (AFB_1_) is ubiquitous in rations intended for poultry consumption, and this molecule possesses the most powerful carcinogenic, teratogenic, mutagenic, and immunosuppressive potential (Rawal et al. 2010). Turkeys are found among the most susceptible species to AFB_1_ due to their deficient detoxification mechanism in the liver (Reed et al. 2019). Consumption of feed contaminated with considerable amounts of AFB_1_ causes various adverse effects in poultry such as alteration in feed consumption, weight gain, increased morbidity and mortality, hematological and biochemical changes, and in some cases reduction in the relative weight of immune organs, as well as important macroscopic and microscopic changes in the liver (Grozeva et al. 2020). Currently, the incidence of AFB_1_ contamination in grains destined for feed production has had greatest variability due to global climate change; consequently, the development of environmentally friendly strategies is required to guarantee the safety of certain feed ingredients (Khodaei et al. 2021). In this context, the most used strategy for the physical control of aflatoxins in feedstuffs is the addition of inorganic adsorbent materials such as zeolites, aluminosilicates, hydrated sodium calcium aluminosilicate (HSCAS), clays, among others. In recent years, plant-based adsorbents have turned out to be promising compared to inorganic adsorbents since they have been shown to be effective and more cost-effective (Vila-Donat et al. 2018).

Several in vitro studies have been carried out using agricultural wastes as adsorbent materials for AFB_1_ removal. In these studies, the main effects of several variables such as pH, temperature, time, and dose (adsorbent/adsorbate) have been evaluated. Very recently, our research group carried out an investigation to determine the effectiveness of an adsorbent made from powdered alfalfa leaves for the removal of AFB_1_ using two in vitro models (Nava-Ramírez et al. 2023). When using a pH-dependent model, adsorption values above 98% were obtained and when an avian intestinal model was utilized, a considerable reduction in the AFB_1_ uptake by the powdered alfalfa leaves was observed (88.8%). However, until now, few in vivo studies have been carried out for the evaluation of the efficacy of plant-based materials as AFB_1_ adsorbents (Gambacorta et al. 2016; Perali et al. 2020; Taranu et al. 2020).

It has been reported that the addition of alfalfa to the diet of poultry significantly improved body weight, body weight gain, growth performance, and the reproductive capacity. Additionally, a positive effect has also been observed in some serum biochemical parameters, and the height of the villi and depth of the duodenal crypt of the intestine (Suwignyo and Sasongko 2019). To the best of our knowledge, the in vivo efficacy of alfalfa as an AFB_1_ adsorbent material has not been reported; consequently, the aim of the present research was to evaluate the effect of powdered alfalfa leaves to ameliorate the toxic effects of AFB_1_ in turkey poults.

## Materials and methods

### Preparation of the adsorbent material

Alfalfa (*Medicago sativa* L.) leaves collected from the botanic garden of the National Autonomous University of Mexico-Superior Studies Faculty at Cuautitlan were washed with distilled water and dried in an oven (Binder model RE-115, Tuttlingen, Germany) at 40 °C for 48 h. The dried leaves were finely ground in an electric plate mill (Glen Mills Inc., Clifton, NJ, USA) and subsequently passed through a 60 mesh to obtain a particle size distribution of < 250 µm. Finally, the powdered alfalfa leaves were deposited in a plastic container and placed in a desiccator over silica pellets. A commercial aflatoxin binder based on yeast cell wall (YCW) from *Saccharomyces cerevisiae* (SafMannan, Phileo Lesaffre Animal Care, Lesaffre Iberica S.A., Valladolid, Spain) was used as a reference material.

### Aflatoxin production

Aflatoxins were produced on rice according to the recommendations of Shotwell et al. (1966). A spore suspension of the *Aspergillus flavus* strain NRRL 2999 was utilized to inoculate the solid substrate in 250-mL Erlenmeyer flasks (50 g of rice + 25 mL water + 0.5 mL of the conidia suspension). Flasks were incubated in a New Brunswick incubator at 28 °C for 5 days under agitation (188 rpm) at the Veterinary Medical Diagnostic Laboratory of the University of Missouri, Columbia, USA. At the end of the incubation period, the aflatoxin-contaminated rice was steam-sterilized (121 °C, 106 kPa, 15 min), dried, finely ground, and the aflatoxin content was estimated by means of liquid chromatography with fluorescence detection according to the recommendations of Göbel and Lusky (2004).

### Preparation of the aflatoxin-contaminated diet and mycotoxin analyses

To assure a proper distribution of the aflatoxins, the highly contaminated rice was previously mixed in the commercial turkey poult diet (Nutricion Tecnica Animal SA de CV, Queretaro, Mexico) to a content of 25,000 ng AFB_1_ per gram of feed. This stock was subsequently used to contaminate the rest of the commercial feed, using 10 g of the stock per kg of feed. The composition of the diet was similar to previously reported data for turkeys (Maguey-González et al. 2023). Batches of 30 kg were artificially contaminated to give a final content of 250 ng AFB_1_/g feed. Before the contamination process, the commercial turkey poult diet was analyzed for total aflatoxins (AFB_1_, AFB_2_, AFG_1_, and AFG_2_), total fumonisins (FB_1_, FB_2_, and FB_3_), and deoxynivalenol (DON) following the recommendations of VICAM, Science Technology, Watertown, MA, USA (https://www.vicam.com/category/aflatoxin-testing-solutions, https://www.vicam.com/category/fumonisin-testing-solutions, and https://www.vicam.com/toxins/deoxynivalenol, accessed on 08 February 2024). The adsorbent materials (powdered alfalfa leaves and YCW) were also mixed in the respective diet at an inclusion level of 0.5% (w/w). Finally, five aflatoxin-contaminated feed samples were taken at random, and the content of AFB_1_ was estimated according to the 991.31 AOAC methodology (Horwitz 2010).

### Experimental design of in vivo experiment

All procedures on experimental animals were in accordance with and approved by the Institutional Animal Care and Use Committee (IACUC) at the University of Arkansas under protocol number 22020. Three hundred and fifty one-day-old female Nicholas-700 turkey poults (Aviagen Inc., AR, USA) were randomly distributed in five pens with seven repetitions each (*n* = 70 per treatment) as follows: (1) Control, AFB_1_-free diet; (2) AF, diet contaminated with 250 ng AFB_1_/g; (3) Alfalfa, AFB_1_-free diet + 0.5% (w/w) adsorbent; (4) AF + alfalfa, diet contaminated with 250 ng AFB_1_/g + 0.5% (w/w) adsorbent, and (5) AF+YCW, diet contaminated with 250 ng AFB_1_/g + 0.5% (w/w) of YCW (a commercial mycotoxin binder used as reference material). Poults were maintained for 28 days with free access to feed and water. The temperature and lighting programs were followed according to the recommendations of the supplier (Aviagen 2015).

### Collection of samples and measurements

Poults and feed were weighed on a weekly basis to calculate the body weight (BW), body weight gain (BWG), feed conversion ratio (FCR), and feed intake (FI). Mortality was recorded throughout the experiment. At the end of the trial, 21 turkeys were randomly selected from each treatment (three poults per replicate) and samples of whole blood were taken and serum prepared. Serum analysis was performed with a spectrophotometer using commercially available kits (BioSystems, Barcelona, Spain) to determine total protein (TP), glucose (Glu), calcium (Ca), uric acid (UA), creatinine (CRE), blood urea nitrogen (BUN), and the enzymes alanine aminotransferase (ALT), alkaline phosphatase (ALP), aspartate aminotransferase (AST), creatine kinase (CK), and glutamate dehydrogenase (GLDH). The determination of hematocrit (Hct), and cell count of leukocytes (WBCs), lymphocytes (Ls), basophils (Ba), monocytes (Mn), and heterophils (H) was performed using an automated hematology analyzer (Cell-Dyn 1700; Abbott Diagnostics, Abbott Park, IL, USA), following the recommendations of Maguey-González et al. (2023). Finally, the bled poults were sacrificed by inhalation of 80% carbon dioxide, 5% oxygen, and 15% nitrogen (Coenen et al. 2000). The liver, spleen, and bursa of Fabricius were removed, rinsed with cold saline, and weighed. For the histological study, liver samples were selected from the left hepatic lobe and fixed in 10% neutral buffered formalin, embedded in paraffin, cut into 5-μm thick sections, and stained with the hematoxylin and eosin (H&E) technique. The slides were examined at 40× magnification. The classification scheme of the semiquantitative evaluation of the liver lesions was as follows: severity grade 0 (lesion not present or within normal levels), grade 1 (mild lesion), grade 2 (moderate lesion), and grade 3 (severe lesion).

### Statistical analysis

Data on productive parameters, relative organ weight, blood count cells, biochemical, and enzymatic analysis were subjected to analysis of variance (ANOVA) using the General Linear Model (GLM) procedure in the Statistical Analysis System software version 8.0 (SAS Institute Inc. Cary, NC, USA). Means were separated by the Tukey procedure. The Kruskal-Wallis non-parametric test was performed to assess the histological analysis. A value of *p* < 0.05 was considered to reject null hypothesis.

## Results and discussion

### Analysis of dietary aflatoxins

In general, the uncontaminated commercial feed samples analyzed contained slightly higher than the lowest detectable content of the total aflatoxins, total fumonisins, and deoxynivalenol. The contents of these fungal toxins in the commercial diet were 1.7 ng/g, 0.02 mg/kg, and 0.09 mg/kg, respectively. Thus, the presence of these mycotoxins in the feed was considered to be negligible (Méndez-Albores et al. 2005). Moreover, the artificially aflatoxin-contaminated turkey diet contained 250 ± 14 ng AFB_1_/g, analyzed by means of the 991.31 AOAC methodology.

### Production parameters

The production parameters of the turkey poults from day 1 to day 28 are shown in Table 1. At the beginning of the experiment (day old poults), no statistically significant differences were found between the five treatments in terms of BW. However, in the last week of the trial (28 day old), it was observed that turkeys that received the diet contaminated with AFB_1_ had a significant reduction in BW, showing a deviation of −12.94%, compared to the Control group. Oyegunwa et al. (2021) reported that offering an experimental diet contaminated with 200 ng AFB_1_/g feed during a 28-day period produced a significant decrease on turkey BW (up to 33% compared to the control group). Other authors also reported a significant reduction in BW in broiler chickens fed a diet contaminated with 200 ng AFB_1_/g (Tessari et al. 2006). On the contrary, the marked reduction in BW caused by the AFB_1_ consumption, improved significantly (*p* < 0.0001) with the use of powdered alfalfa leaves; consequently, poults of the AF+alfalfa group presented a deviation of − 4.4% compared to the Control group. Furthermore, with the use of YCW, a significant reduction in the BW was also observed in the AF+YCW group showing a deviation of −10.2% compared to the Control group. These results agree with the findings reported by Hernández-Ramírez et al. (2021), who reported that the addition of YCW (0.05%) to a diet contaminated with AFB_1_ (500 ng/g) did not alleviate the negative effects caused by the mycotoxin in broiler chickens. Interestingly, the treatment that was provided with the powdered alfalfa leaves (Alfalfa) had a significantly higher BW, up to 528.21 g/poult (7.41 deviation compared to the Control group). Ouyang et al. (2016) also reported that the inclusion of alfalfa flavonoids (15 mg/kg) in a diet intended for female broilers significantly improved BW by 4.77%. These results are consistent with our findings.

**Table 1:** Evaluation of body weight (BW), body weight gain (BWG), feed intake (FI), and feed conversion ratio (FCR) in 28-day-old turkey poults consuming a maize-soybean based diet contaminated with 250 ng AFB_1_/g supplemented with the powdered alfalfa leaves and yeast cell wall

Treatment	Body weight (g)		Body weight gain (g)		Feed intake (g)	Feed conversion ratio
		Deviation from Control (%)				
Control	491.76 ± 8.32^a,b^	0	435.00 ± 8.20^a,b^	683.68 ± 25.10^a,b^		1.571 ± 0.02
AF	428.14 ± 6.71^c^	-12.94	371.76 ± 6.74^c^	567.76 ± 7.00^c^		1.533 ± 0.04
Alfalfa	528.21 ± 3.83^a^	7.41	471.56 ± 3.70^a^	726.27 ± 23.57^a^		1.532 ± 0.04
AF+alfalfa	470.17 ± 9.81^b,c^	-4.40	414.61 ± 9.94^b,c^	607.30 ± 22.89^b,c^		1.474 ± 0.04
AF+YCW	441.68 ± 10.38^c^	-10.18	385.30 ± 10.64^c^	584.60 ± 34.70^c^		1.512 ± 0.03
*p*-value	< 0.0001		< 0.0001	< 0.001		0.16

Seven replicates per group (n = 10 poults per replicate). Means ± SE

^abc^Means with non-matching superscripts within columns indicates significant difference (*p* < 0.05)

Regarding BWG, turkeys that were given the AF-free diet with the addition of powdered alfalfa leaves had no statistically significant differences when compared to the Control group (471.56 vs 435.00 g). However, the three experimental groups that were fed with the diet contaminated with AFB_1_ (AF, AF+alfalfa, and AF+YCW), showed a significant reduction in BWG compared to the Control and Alfalfa groups. These results are similar to those reported by Samur et al. (2020), who showed that the supplementation of a commercial duckling diet with 10% (w/w) fresh alfalfa had no effect in BWG. Interestingly, poults that were given the diet supplemented with powdered alfalfa leaves had the greatest numerical value in BWG compared to the Control group (but not statistically significant). In this context, Suwignyo et al. (2021) showed that 3% (w/w) alfalfa supplementation in the diet increased BWG up to 8.05% in ducks from 1 to 35 days of age. In this research, the AF+alfalfa group did not present a significant difference in BWG compared to the Control group, and this could possibly be explained by the immuno- and hepatoprotective effect of the powdered alfalfa leaves. Moreover, a significant reduction in FI (g/poult) was observed in the AF and AF+YCW groups compared to the Control group, attaining a reduction of up to −16.96% and −14.49%, respectively (Table 1). These results agree to those reported by Rauber et al. (2007), who showed a −9.13% reduction in FI in turkeys fed a diet with 200 ng AFB_1_/g feed. Additionally, Singh (2019) reported a significant decrease (up to −34.61%) in feed consumption in 4-week-old turkey poults, which were fed a diet with 150 μg AFB_1_/kg feed. In this work, there was no significant difference in FI between the AF+alfalfa and the Control group (Table 1). Furthermore, it was observed that FI was significantly higher in the group of turkeys that were offered the AFB_1_-free diet with the addition of the powdered alfalfa leaves (Table 1). In this context, Suwignyo et al. (2021) also showed that 3% (w/w) alfalfa supplementation in the diet increased FI and BWG of ducks at 35 days of age. In this study, it was observed that there was no statistically significant difference in the FCR between treatments. These results are in close agreement with Samur et al. (2020), who reported that the consumption of a diet contaminated with 200 ng AFB_1_/g feed does not show a significant difference in FCR in turkeys. Finally, the mortality observed during the experiment (7%) was not related to the dietary AFB_1_ level, but rather due to yolk sac infection.

### Relative weight of organs

The effect of the different treatments on the relative organ’s weight is shown in Table 2. Compared to the Control group, the relative weight of the liver decreased significantly in the poults of the four experimental groups (AF, Alfalfa, AF+alfalfa, and AF+YCW). These results are consistent to those reported by Gómez-Espinosa et al. (2017), who observed that the relative weight of the liver of the poults that consumed a diet contaminated with AFB_1_ (331 ng AFB_1_/g feed) decreased significantly. Moreover, the group of turkeys that were offered the AFB_1_-free diet with the addition of powdered alfalfa leaves (Alfalfa) and the AF+alfalfa group did not show significant differences in terms of the relative weight of the spleen compared to the Control group (Table 2). Notoriously, two of the experimental groups that were fed with the diet contaminated with AFB_1_ (AF and AF+YCW), showed a significant increase on the relative weight of the spleen compared to the Control group (Table 2). Peng et al. (2015) showed that broiler chickens that were fed with a diet contaminated with aflatoxins (216.4 ng AFB_1_/g feed) increased the relative weight of the spleen. Finally, the three groups that were fed with the diet contaminated with AFB_1_ showed that the relative weight of the bursa of Fabricius increased by 11.1% (AF), 11.1% (AF+alfalfa), and 16.7% (AF+YCW), with respect to the Control group. Regarding the bursa/spleen ratio, there were no statistically significant differences between treatments. Maguey-González et al. (2023) reported that the relative weight of the spleen and the bursa of Fabricius increased significantly in poults that were fed with a diet containing AFB_1_ (250 ng AFB_1_/g feed). These results are in accordance with those obtained in the present study.

**Table 2:** Relative weight of the liver, spleen, bursa of Fabricius in 28-day-old turkey poults consuming a maize-soybean based diet contaminated with 250 ng AFB_1_/g supplemented with powdered alfalfa leaves and yeast cell wall

Treatment	Liver	Spleen	Bursa of Fabricius	Bursa/spleen ratio
Control	3.72 ± 0.133^a^	0.11 ± 0.005^b^	0.18 ± 0.010^b^	1.56 ± 0.095
AF	2.89 ± 0.127^b^	0.17 ± 0.009^a^	0.20 ± 0.010^a,b^	1.18 ± 0.069
Alfalfa	3.08 ± 0.121^b^	0.12 ± 0.005^b^	0.16 ± 0.007^b^	1.42 ± 0.081
AF+alfalfa	2.89 ± 0.134^b^	0.14 ± 0.009^a,b^	0.20 ± 0.012^a,b^	1.43 ± 0.115
AF+YCW	3.08 ± 0.119^b^	0.16 ± 0.011^a^	0.21 ± 0.011^a^	1.40 ± 0.120
*p*-value	< 0.0001	< 0.0001	0.0042	0.10

Means ± SE

^ab^Means with non-matching superscripts within columns indicates significant difference (*p* < 0.05)

### Blood count cells

At the end of the trial, certain trends were observed in the blood cell count values; however, no significant differences were found in all the values analyzed such as Hct, WBCs, Ls, the ratio of H to Ls, Ba, Mn, and H (Table 3). These results agree with what was reported by Quist et al. (2000) and Oyegunwa et al. (2021), who studied the effect of a diet contaminated with 200 and 150 ng AFB_1_/g in turkey poults, respectively.

**Table 3:** Blood count cells in 28-day-old turkey poults consuming a maize-soybean based diet contaminated with 250 ng AFB_1_/g supplemented with powdered alfalfa leaves and yeast cell wall

Treatment	Hct	WBCs	H	Ls	H/L	Ba	Mn
Control	40.75 ± 3.22	15.87 ± 1.67	6421 ± 1505.83	6966 ± 492.59	0.98 ± 0.29	961 ± 298.73	1500 ± 284.19
AF	36.60 ± 3.36	20.58 ± 5.07	5169 ± 1592.10	6749 ± 1013.02	0.73 ± 0.12	650 ± 154.08	2423 ± 669.84
Alfalfa	45.66 ± 0.84	16.59 ± 1.93	5950 ± 657.23	7286 ± 890.40	0.85 ± 0.07	433 ± 203.19	2918 ± 491.72
AF+alfalfa	42.80 ± 2.54	14.26 ± 2.10	5720 ± 1018.31	5942 ± 777.85	0.94 ± 0.09	216 ± 122.07	2363 ± 792.75
AF+YCW	37.50 ± 2.20	19.90 ± 1.41	6627 ± 764.86	8795 ± 999.97	0.83 ± 0.12	784 ± 293.27	3783 ± 720.88
*p*-value	0.097	0.46	0.90	0.22	0.77	0.16	0.19

Means with non-matching superscripts within columns indicates significant difference (*p* < 0.05). Seven replicates per group (n = 3 poults per replicate). Means ± SE

*Hct* Hematocrit, *WBCs* Leukocytes, *Ls* Lymphocytes, *H/L* ratio of heterophils to lymphocytes, *Ba* Basophils, *Mn* Monocytes, *H* heterophils

### Selected biochemical constituents

Table 4 shows the results of some biochemical constituents. In general, the plasmatic concentrations of TP, Glu, Ca, CRE, and BUN in the poults fed with the AFB_1_-contaminated diet significantly decreased compared to the Control group. Aflatoxin consumption by poultry has been reported to cause a reduction in serum total protein levels due to impaired protein synthesis in the liver (Van Rensburg et al. 2006). Thus, 2.27-, 1.91-, and 2-fold decrease in total serum protein was found in the AF, AF+alfalfa, and AF+YCW groups, respectively. Finally, the group of poults fed the AFB_1_-free diet, and the Alfalfa group did not present a significant difference in the TP concentration compared with the Control group. These findings are in accordance with earlier reports, Wan et al. (2013) reported that the amount of TP in the serum of ducks that consumed a diet contaminated with 100 ng AFB_1_/g feed, decreased by 1.13-fold.

**Table 4:** Biochemical parameters in 28-day-old turkey poults consuming a maize-soybean based diet contaminated with 250 ng AFB_1_/g supplemented with powdered alfalfa leaves and yeast cell wall

Treatment	TP	Glu	Ca	UA	CRE	BUN
Control	3.82 ± 0.10^a^	583.00 ± 49.90^a^	9.04 ± 0.35^a^	10.80 ± 0.23	0.20 ± 0.01^a^	4.6 ± 0.50^a,b^
AF	1.68 ± 0.04^b^	405.00 ± 12.84^b,c^	5.76 ± 0.29^b^	9.74 ± 0.25	0.11 ± 0.01^b^	3.6 ± 0.24^b^
Alfalfa	3.88 ± 0.09^a^	528.20 ± 31.22^a,b^	8.58 ± 0.35^a^	10.10 ± 0.20	0.23 ± 0.01^a^	4.6 ± 0.24^a,b^
AF+alfalfa	2.00 ± 0.08^b^	373.00 ± 18.89^c^	5.74 ± 0.29^b^	8.50 ± 0.21	0.12 ± 0.01^b^	4.8 ± 0.20^a^
AF+YCW	1.90 ± 0.08^b^	382.20 ± 17.79^c^	4.66 ± 0.24^b^	9.24 ± 0.26	0.13 ± 0.01^b^	4.8 ± 0.20^a^
*p*-value	< 0.001	0.0003	< 0.0001	0.34	< 0.0001	< 0.001

Means ± SE

*TP* Total protein, *Glu* Glucose, *Ca* Calcium, *UA* uric acid, *CRE* creatinine, *BUN* blood urea nitrogen

^abc^Means with non-matching superscripts within columns indicates significant difference (*p* < 0.05). Seven replicates per group (n = 3 poults per replicate)

In general, the groups of turkeys that were fed a diet contaminated with AFB_1_ (AF, AF+alfalfa, and AF+YCW) showed a decrease in serum Glu, Ca, and CRE concentrations, compared to the Control group (Table 4). On the other hand, the group of turkeys that were offered the AFB_1_-free diet but with the addition of the powdered alfalfa leaves, did not show significant differences in the serum concentration of Glu, Ca, and CRE. These findings agree with various reports with broilers (Gowda et al. 2009; Sridhar et al. 2015; Gómez-Espinosa et al. 2017; Hernández-Ramírez et al. 2021). On the other hand, there were no significant differences in the concentration of UA. Sharma et al. (2019) showed that the consumption of a diet contaminated with 150 and 300 ng AFB_1_/g feed in broiler chickens did not affect the UA concentration. Finally, the BUN value decreased only in the AF group. In this context, Xie et al. (2022) showed that the BUN value was not affected in broilers fed with two levels of AFB_1_ (60 and 120 ng/g), at 21 and 42 days post-exposure.

### Liver enzymes

Table 5 shows the results of some serum liver enzymes. There were no statistically significant differences between the five experimental groups in the serum concentrations of ALP, AST, CK, and GLDH. Comparable results are also reported by other researchers (Quist et al. 2000; Gómez-Espinosa et al. 2017). However, the only enzyme in which a marked increase was observed in the AF+YCW group was the ALT. Lala et al. (2016) reported that ALT levels showed a significant increase in poultry fed with 60 ng AFB_1_/g feed, which is consistent with our findings.

**Table 5:** Liver enzyme concentrations in 28-day-old turkey poults that consumed a maize-soybean based diet contaminated with 250 ng AFB_1_/g supplemented with powdered alfalfa leaves and yeast cell wall

Treatment	ALT	ALP	AST	CK	GLDH
Control	4.80 ± 1.11^b^	2569.40 ± 130.07	302.60 ± 15.38	6644 ± 1567.32	11.00 ± 1.26
AF	3.00 ± 1.00^b^	2561.40 ± 123.31	259.00 ± 13.61	3832 ± 474.72	8.00 ± 0.44
Alfalfa	4.60 ± 1.66^b^	2471.40 ± 127.44	298.80 ± 25.25	5032 ± 2141.89	17.60 ± 6.09
AF+alfalfa	2.20 ± 0.20^b^	2159.20 ± 66.77	247.20 ± 14.01	3460 ± 738.57	7.6 ± 1.53
AF+YCW	8.80 ± 2.83^a^	2356.80 ± 119.92	318.80 ± 36.72	10244 ± 742.04	7.4 ± 1.12
*p*-value	< 0.001	0.43	0.11	0.13	0.14

Seven replicates per group (n = 3 poults per replicate). Means ± SE

*ALT* alanine transaminase, *ALP* alkaline phosphatase, *AST* aspartate aminotransferase, *CK* creatine kinase, *GLDH* glutamate dehydrogenase

^ab^Means with non-matching superscripts within columns indicates significant difference (*p* < 0.05)

### Liver histology

**Fig. 1:**
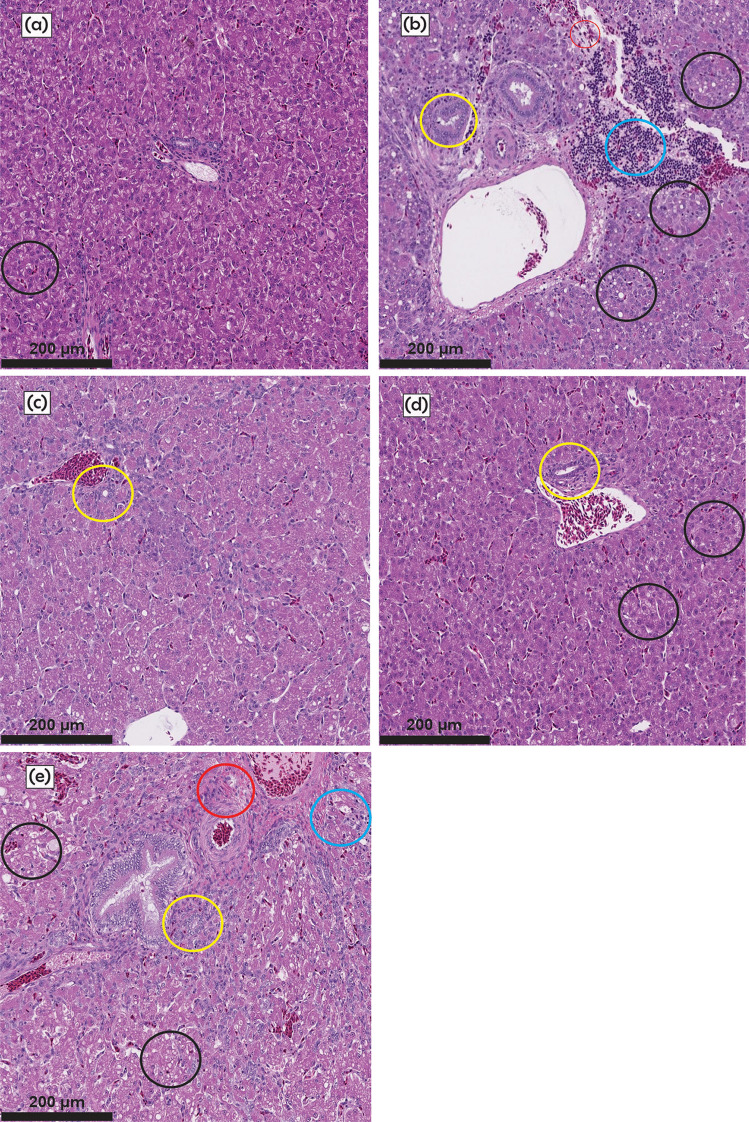
Comparative histological changes in the liver of 28-day-old turkey poults that consumed a maize-soybean based diet contaminated with 250 ng AFB_1_/g and supplemented with powdered alfalfa and yeast cell wall. H&E-stained tissue of: **a** Control, **b** AF, **c** Alfalfa, **d** AF+alfalfa, and **e** AF+YCW. Black circles show vacuolar degeneration, blue circles show inflammation, yellow circles show bile duct hyperplasia, and red circles show fibrosis

Table 6 and Fig. 1 show the histopathological changes in the liver of turkey poults. In general, no significant liver lesions were observed in the Control group (Fig. 1a). However, exposure to AFB_1_ caused important changes in the liver of poults. For instance, a significant increase in the severity of liver lesions, including vacuolar degeneration, inflammation, bile duct hyperplasia, and fibrosis was observed in the AF group (Fig. 1b). Previous studies have reported histopathological alterations in the liver of poultry fed with AFB_1_ levels ranging from 50 to 1000 ng AFB_1_/g (Giambrone et al. 1985; Sridhar et al. 2015; Lala et al. 2016; Hernández-Ramírez et al. 2021; Xie et al. 2022). These alterations include vacuolar degeneration, fatty liver, hemorrhage, congestion, leukocyte infiltration, bile duct hyperplasia, hypertrophy, and portal fibrosis. Conversely, in the two experimental groups supplemented with the powdered alfalfa leaves, no significant differences in the liver lesions were observed compared to the Control group (Table 6), suggesting that the lesions caused by the AFB_1_ were significantly ameliorated when the powdered alfalfa was included in the AFB_1_-contaminated diet. Regarding the AF+YCW group, it was observed that there were no significant differences in inflammation and hyperplasia of bile ducts, compared with the Control group. However, marked vacuolar degeneration and fibrosis were observed (Fig. 1e). In general, the decrease in lesions in poults that received 0.5% (w/w) powdered alfalfa could be related to the effects of flavonoids, carotenoids, and chlorophylls contained in the leaves and mainly to its adsorption properties against AFB_1_ (Nava-Ramírez et al. 2023).

**Table 6:** Histopathological changes in the liver of 28-day-old turkey poults that consumed a maize-soybean based diet contaminated with 250 ng AFB_1_/g supplemented with powdered alfalfa leaves and yeast cell wall

Treatment	Vacuolar degeneration	Inflammation	Bile duct hyperplasia	Fibrosis
Control	1.00 ± 0.28^b^	0.50 ± 0.20^b^	0^b^	0^b^
AF	2.00 ± 0.28^a^	2.00 ± 0.22^a^	2.00 ± 0.15^a^	1.00 ± 0.18^a^
Alfalfa	1.00 ± 0.20^b^	0^b^	1.00 ± 0.11^b^	0^b^
AF+alfalfa	1.50 ± 0.27^a,b^	1.00 ± 0.14^a,b^	1.50 ± 0.09^b^	0^b^
AF+YCW	2.00 ± 0.24^a^	1.00 ± 0.23^a,b^	1.50 ± 0.18^b^	1.00 ± 0.11^a^
***p***-value	< 0.01	< 0.0001	< 0.001	< 0.0001

Seven replicates per group (n = 3 poults per replicate). Means ± SE

^ab^Means with non-matching superscripts within columns indicates significant difference (*p* < 0.05)

To the best of our knowledge, this is the first report showing the effect of a low inclusion level (0.5%, w/w) of powdered alfalfa leaves in the diet of turkey poults contaminated with AFB_1_ (250 ng/g) on production parameters, relative organ weights, total blood cell counts, biochemical and enzymatic parameters, and liver histology. In general, the addition of the adsorbent material derived from powdered alfalfa leaves significantly improved the production parameters, the relative weight of the organs, and the histological lesions of the liver. Moreover, the powdered alfalfa leaves did not cause deleterious changes in the biochemical and enzymatic parameters, as well as in the total blood cell count. Taken together, these results allowed us to confirm that powdered alfalfa is an effective material to adsorb AFB_1_ and, therefore, prevent its toxic effects in in vivo trials. However, it is pertinent to carry out more in vivo studies on the complete growing cycle of this and other avian species to understand the complete protective role of powdered alfalfa leaves and other plant-based materials with adsorptive properties against AFB_1_. Research in this direction is in progress in our laboratories.

## Data Availability

The datasets generated during and/or analyzed during the current study are available from the corresponding author on reasonable request.
